# Microbiome-Derived Trimethylamine N-Oxide (TMAO) as a Multifaceted Biomarker in Cardiovascular Disease: Challenges and Opportunities

**DOI:** 10.3390/ijms252312511

**Published:** 2024-11-21

**Authors:** Kinga Jaworska, Wojciech Kopacz, Mateusz Koper, Marcin Ufnal

**Affiliations:** Laboratory of Centre for Preclinical Research, Department of Experimental Physiology and Pathophysiology, Medical University of Warsaw, Banacha 1B, 02-091 Warsaw, Poland; wojtekkop1@gmail.com (W.K.); mateuszkoper988@gmail.com (M.K.); mufnal@wum.edu.pl (M.U.)

**Keywords:** trimethylamine oxide, biomarker, cardiovascular marker, bacterial metabolites, surrogate marker

## Abstract

Biomarkers play a crucial role in various stages of disease management, including screening, diagnosis, prediction, prognosis, treatment, and safety monitoring. Although they are powerful tools in disease diagnosis, management, and drug development, identifying and validating reliable biomarkers remains a significant challenge. Among potential microbiome-derived biomarkers, trimethylamine N-oxide (TMAO) has gained notable attention for its link to atherosclerosis and cardiovascular risk. However, despite the growing body of research on TMAO, its practical application in clinical settings for disease management and patient outcome enhancement is still not a reality. This paper presents recent data on the utility of TMAO as a cardiovascular biomarker, categorized by its various roles: diagnostic, prognostic, susceptibility/risk, monitoring, pharmacodynamic/response, predictive, and safety. It also briefly discusses research on TMAO’s potential role in cardiovascular disease development. While TMAO shows promise, particularly in prognostic applications, its reliability as a biomarker has been inconsistent across studies. These variances may result from several confounding factors that affect TMAO plasma levels, including diet, kidney function, and demographic variables. The review aims to elucidate the specific contexts in which TMAO can be valuable, potentially leading to more personalized and effective management of cardiovascular disease.

## 1. Introduction

Recent decades have witnessed a surge in research activity focused on biomarkers. As defined by the FDA and NIH in the Biomarkers, EndpointS, and other Tools (BEST) Resource, a biomarker is a defined characteristic measured as an indicator of normal biological processes, pathogenic processes, or responses to exposure or intervention, including therapeutic interventions [1]. In practical terms, biomarkers are tools and technologies designed for various functions corresponding to different disease stages: screening, diagnosis, prediction, prognosis, treatment monitoring, and addressing safety concerns. While offering a powerful approach in disease management and drug development, identifying and properly validating biomarkers remains a challenge. Validation involves establishing if a biomarker’s performance is acceptable for its intended purpose (fit-for-purpose), i.e., whether it measures or predicts the clinical concept of interest [1]. There are many quantitative measures of biomarker performance such as sensitivity, specificity, positive and negative predictive values, likelihood ratios, receiver operating characteristic curve, etc. Actually, the desired properties of a biomarker may vary depending on its intended use. Either way, a clinically valuable biomarker has to significantly add to a patient assessment independently of already established predictors [2]. Its use should translate into improved patient outcomes. Therefore, while many biomarker candidates appear promising, very few advance into widespread clinical practice.

Abundant evidence indicates a robust mutual relationship between the gut microbiome and its host, encompassing cardiovascular and metabolic regulation [3,4]. Consequently, evaluating ‘microbial status’ or bacterial-origin compounds emerges as a potential tool for enhancing diagnostic accuracy and therapeutic response. Significantly, the rapid advancement in metagenomics and metabolomics has introduced numerous potential biomarker candidates, such as short-chain fatty acids and taurine [5]. In the context of the circulatory system, microbiome-derived trimethylamine N-oxide (TMAO) has garnered significant attention due to its association with atherosclerosis burden [6] and cardiovascular risk [7].

## 2. TMAO Metaorganismal Pathway

**Figure 1:**
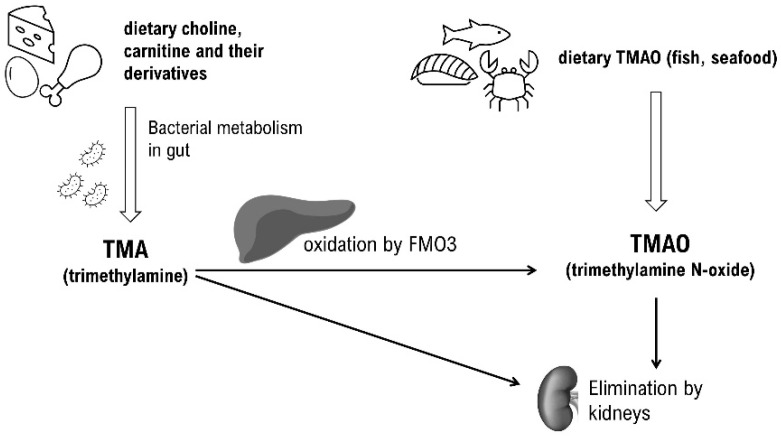
TMA/TMAO metaorganismal pathway. TMA—trimethylamine; TMAO—trimethylamine N-oxide; FMO3—flavin-containing monooxygenase 3.

The human body generates TMAO through two primary pathways. The first involves the metabolism of dietary choline, carnitine, and their derivatives by the gut microbiome into trimethylamine (TMA). This metabolic process is facilitated by specific bacterial enzymes known as choline TMA lyases (CutC). TMA then crosses the gut–blood barrier, entering the portal circulation. In the liver, most of the TMA is oxidized into TMAO by the enzyme hepatic flavin-containing monooxygenase (FMO3). The second pathway is the direct intake of TMA and TMAO, which are abundantly found in fish and other seafood [8]. In fact, consuming fish may result in a greater increase in circulating TMAO levels compared to foods rich in choline and carnitine [9]. The major route of TMAO elimination is via the kidneys but to a small extent it can also be excreted in sweat and breath [10]. It is important to emphasize that the tissue distribution and kinetics of methylamines in the blood are largely unknown, and various factors must be considered when assessing TMAO levels. First, the absorption of methylamines in the intestine is influenced by diet, as well as the composition and metabolic activity of the gut microbiota. Additionally, the rate of TMA oxidation is contingent on the activity of the enzyme FMO3, which can vary based on sex, race, genetic polymorphisms, or the overall condition of the liver [11,12]. Lastly, kidney function, particularly the rate of excretion, significantly influences TMAO concentration [13]. These factors, along with others, lead to relatively high inter- and intra-individual variations [14] that can significantly impede the utility of TMAO as a biomarker (Figure 1).

## 3. TMAO as a Biomarker

Plasma TMAO has been suggested as a potential biomarker in cardiovascular disease. However, the robustness of TMAO as a biomarker has yielded conflicting results in various studies. These discrepancies may be partly due to numerous confounding factors that influence TMAO plasma levels, such as kidney function, diet, and demographic variables. In this paper, we present an overview of the latest data on the utility of TMAO as a biomarker, ensuring clarity and consistency in terminology based on definitions from the BEST Resource [2].

### 3.1. Diagnostic Biomarker

Diagnostic biomarkers are tools used to detect or confirm the presence of a disease (or its subtype) or other condition for which treatment may be indicated [2]. Elevated plasma TMAO levels have recently been associated with various cardiovascular disorders, including acute ischemic stroke [15,16,17], coronary artery disease [18], heart failure [19,20,21], and renal dysfunction [19] (Table 1). Additionally, several studies have indicated that TMAO levels may vary with the severity of these conditions. For instance, TMAO has shown diagnostic utility in distinguishing critical limb ischemia among patients with peripheral artery disease [22] and its levels correlate with the severity of coronary atherosclerosis in acute coronary syndrome patients [23,24,25]. Furthermore, in patients with ischemic stroke, TMAO was suggested to differentiate between mild and moderate to severe strokes [17] and identifying early neurological deterioration [26]. TMAO has also been explored for its potential in detecting complications in cardiovascular patients, such as thrombus formation in atrial fibrillation [27], kidney disease in hypertensive patients [28], and subclinical myocardial damage in high-risk patients [29]. It may even detect subclinical atherosclerosis in children with obesity [30]. While some studies have shown statistical differences in TMAO concentrations between affected and unaffected individuals, they often lack assessment using standard diagnostic methods like receiver operator characteristic curves [31,32,33,34,35]. Conversely, certain studies have not confirmed the diagnostic accuracy of TMAO in conditions like coronary artery disease [36] or atrial fibrillation [37].

Currently, there is no conclusive evidence that evaluating plasma TMAO levels significantly enhances established clinical assessments. For example, combining TMAO measurements with N-terminal prohormone of brain natriuretic peptide (NT-proBNP) yielded similar diagnostic results to NT-proBNP alone in heart failure patients with preserved ejection fraction [21].

Therefore, the data on the diagnostic utility of TMAO are promising and its validation as a diagnostic biomarker in the near future calls for further research.

### 3.2. Prognostic Biomarker

Prognostic biomarkers are employed to determine the likelihood of specific outcomes, such as future clinical events, disease recurrence, or progression, in populations with a disease [2]. Generally, assessing their usefulness requires a prospective study and a larger sample size than that needed for diagnostic biomarkers. In the case of TMAO, studies commonly report the odds or hazard ratios for specified outcomes, like all-cause mortality or major cardiac events, based on the quartiles of TMAO levels. The prognostic utility of TMAO has been suggested in conditions such as acute ischemic stroke, heart failure, peripheral and coronary artery disease, chronic kidney disease, among others (see Table 2 for references). Observations have linked high-plasma TMAO with long-term outcomes [38,39,40], and several studies have demonstrated its incremental prognostic value in conventional risk stratification [31,39,41,42,43]. For instance, incorporating TMAO into a model of traditional cardiovascular risk factors significantly improved the 5-year all-cause mortality risk assessment in stable coronary artery disease patients [42]. It has been postulated that measuring TMAO may be particularly useful in patients with heart failure with preserved ejection fraction (HFpEF), where natriuretic peptide concentrations are relatively lower than in heart failure with reduced ejection fraction (HFrEF), making risk stratification more challenging [44]. Research suggests that, in addition to hard clinical endpoints, plasma TMAO levels may also predict poor functional outcomes in stroke patients [31,45]; however, the data are not consistently supportive [32] (see Table 2). Unfortunately, many cited studies evaluating the prognostic value of plasma TMAO level did not adjust for estimated glomerular filtration rate (eGFR), i.e., renal function. It should be highlighted that low eGFR is a well-established risk factor for cardiovascular mortality [46,47]. In this context, many studies that included eGFR adjustment in their statistical model showed partial or complete attenuation of the associations between TMAO and adverse outcomes [43,48,49,50,51,52,53,54]. Moreover, several studies have linked TMAO to certain study endpoints but not others [32,55,56]. For example, TMAO was associated with cardiovascular death but not with major adverse cardiac events [56]. A study by Li et al. underscores the critical importance of timing in assessing TMAO concentrations, showing that only patients with high TMAO levels at both initial admission and at a one-month follow-up exhibited an increased risk of major adverse cardiovascular events (MACE) [57]. Future research should focus on identifying the patient populations, timepoints, and outcomes where circulating TMAO may have the most promising prognostic performance. Lastly, it remains unclear whether TMAO is a modifiable biomarker. Currently, there is scant clinical data on the effect of treatment on TMAO concentration (discussed further in Section 3.5, Pharmacodynamic/Response Biomarker).

In conclusion, most of the studies point to TMAO’s prognostic utility in various cardiovascular diseases; however, there is some inconsistency, probably due to variances in kidney function, patient populations, and timepoints of TMAO assessment.

### 3.3. Susceptibility/Risk Biomarker

A susceptibility/risk biomarker is one that indicates the potential for developing a disease or medical condition in individuals with no clinically apparent disease [2]. Conceptually, assessing TMAO could identify a population particularly susceptible to cardiovascular disease and encourage more aggressive modification of risk factors. There are a few studies that have evaluated the prognostic potential of TMAO in the general population. Zheng et al. prospectively investigated the interaction between circulating TMAO and future risk of incident cardiovascular disease in a sample from community-based Chinese adults. TMAO was associated with a higher risk, and analysis confirmed that a model including TMAO had better discrimination than one containing only traditional risk factors [97]. Two other studies on population-based cohorts, one Chinese and one US, revealed the association of TMAO with a higher stroke risk [98,99]. Similarly, TMAO provided incremental risk prediction for future coronary artery disease in the community-based EPIC-Norfolk population [100]. Other Norwegian cohorts have shown that plasma levels of TMAO are positively associated with a risk of incident atrial fibrillation [101]. Conversely, the prospective PREDIMED study on participants at high cardiovascular risk revealed that TMAO level was not associated with a risk of atrial fibrillation or heart failure [102]. Several other studies also do not support the role of TMAO as a susceptibility/risk biomarker. In a prospective nested case–control study conducted among individuals without diabetes, cardiovascular disease, or cancer, there was no association between TMAO and the risk of coronary artery disease [103]. Similarly, a cross-sectional study on elderly Japanese adults showed that TMAO was not a risk factor for atherosclerosis in this population [104]. In addition, in a large cohort of older US adults, a significant interaction between TMAO and the risk of future cardiovascular disease was found only in patients with impaired kidney function [105,106]. Incidentally, a recent study by Heianza et al. in the Nurses’ Health Study cohort showed that a 10-year increase in TMAO concentration corresponded to an increased risk of coronary heart disease, regardless of the initial level of TMAO and other conventional factors [107].

On the whole, data are contradictory, and the utility of TMAO as a risk biomarker in subjects with no clinically apparent disease requires further investigation.

### 3.4. Monitoring Biomarker

A monitoring biomarker is one that is measured repeatedly and is generally used to assess disease progression, including the occurrence of new disease effects, response to treatment, or evidence of exposure to an environmental agent [2]. Currently, there are no data specifically evaluating TMAO performance as a monitoring biomarker per se, but several studies have provided repeated measurements of TMAO. For instance, the study by Heianza et al., which was already mentioned, aimed to assess the association between temporal changes in plasma TMAO and the incidence of coronary heart disease. Therefore, they measured TMAO at two time points, approximately 10 years apart [107]. In another case, there was inconsistency in data regarding TMAO levels in stroke patients, and two studies assessed fluctuations in TMAO by determining its concentration at three different time points [32,47]. Specifically, they revealed that TMAO levels decreased significantly 48 h after stroke onset and increased again three months later.

All in all, current data do not provide sufficient evidence to evaluate TMAO performance as a monitoring biomarker.

### 3.5. Pharmacodynamic/Response Biomarker

A pharmacodynamic/response biomarker demonstrates a biological response to treatment or exposure to an environmental agent. Variations in this biomarker type offer preliminary evidence of a treatment’s effect on a desired clinical endpoint. It also provides insights into whether to continue a treatment or adjust the dosage [2]. Numerous animal studies have indicated the causative role of TMAO in cardiovascular pathology [6,108,109]. TMAO is postulated as a mediator linking disturbances in gut microbiota (i.e., dysbiosis) with adverse clinical outcomes. Accordingly, various studies have aimed at modifying the intestinal microbiome to subsequently lower TMAO levels [6,110]. In this context, TMAO can be considered a candidate pharmacodynamic/response biomarker, especially in microbiota-targeted therapies. However, human research in this area is scant. Early results from a small randomized placebo-controlled study suggest that polyphenols may reduce TMAO concentration [111]. Moreover, Park et al. conducted a dietary intervention study, observing a significant increase in TMAO levels during short-term popular diets [112]. In contrast, prolonged, high-dose probiotic supplementation did not alter TMAO concentrations [113,114,115,116] nor did treatment with rifaximin [117]. TMAO was also evaluated in studies assessing response to oral l-carnitine therapy in mitochondrial disorders [118,119]. While these studies presuppose TMAO’s pathological role, the data are conflicting and warrant further investigation [120].

As a potential response biomarker in cardiovascular diseases, the modifiability of TMAO levels by guideline-recommended therapy remains uncertain. The BIOSTAT-CHF study showed that standard heart failure treatment does not affect TMAO levels [57]. In patients with symptomatic heart failure, TMAO levels stayed elevated over the long term, even after left ventricular assist device implantation and heart transplant [121]. Conversely, Yang et al. observed a dynamic decrease in TMAO levels among stable and improved patients with pulmonary hypertension following treatment, but an upward trend among those with worsened risk status [122]. Data regarding the impact of statins and Sodium–Glucose Transport Protein 2 (SGLT2) inhibitors on TMAO concentrations are contradictory. Some studies suggest a causal relationship between statins and decreased TMAO levels [123] with statin use inversely associated with TMAO levels in adults at risk for atherosclerosis [124]. However, long-term statin therapy in diabetic patients did not affect plasma TMAO levels [125]. Experimental studies have suggested the inhibitory effect of SGLT2 inhibitors on TMAO precursors [126], but a secondary analysis of the EMMY trial indicated a significant increase in TMAO levels post-myocardial infarction in patients receiving Empagliflozin compared to those on standard treatment [127].

Therefore, the conflicting results underscore the need for further research into the effects of cardiovascular disease-modifying drugs on TMAO levels and their predictive value.

### 3.6. Predictive Biomarker

A predictive biomarker is instrumental in identifying individuals who may experience favorable or unfavorable effects from exposure to a treatment or environmental agent. Such biomarkers are commonly utilized to ascertain who might benefit from a specific treatment or to choose among various therapeutic options. Establishing a predictive biomarker necessitates comparing the intervention with a control treatment in individuals both with and without the biomarker [2]. To date, only a limited number of studies have evaluated the influence of TMAO on treatment efficacy. Specifically, Gencer et al. investigated how TMAO impacts the effectiveness of ticagrelor in patients with myocardial infarction. Their findings indicated that the reduction in major adverse cardiac events due to ticagrelor was consistent, irrespective of TMAO levels [75]. Their observation raises questions, especially if TMAO is indeed a mediator in cardiovascular diseases as some theories suggest. The limited experimental data from animal studies seem to support the potential role of TMAO [128], highlighting the need for more extensive research in this area to clarify its impact on therapeutic outcomes.

In conclusion, current data are not sufficient to evaluate TMAO performance as a predictive biomarker.

### 3.7. Safety Biomarker

A safety biomarker is crucial for detecting or predicting adverse effects from drug usage or environmental exposure. Ideally, such a biomarker would indicate the onset of toxicity before clinical symptoms or irreversible damage occur [2]. Currently, there are limited data on using TMAO as a safety biomarker. However, TMAO is recognized as a uremic toxin whose levels significantly increase with kidney function decline [13,58], suggesting its potential utility as a biomarker for renal toxicity. Notably, the direct linear relationship between the eGFR and TMAO levels remains unclear [129]. Additionally, there is a notable lack of data on TMAO concentration changes in cases of acute kidney injury [130]. This gap in knowledge highlights the need for further research to establish TMAO’s efficacy and reliability as a safety biomarker, particularly in renal toxicity contexts.

To sum up, there are limited data on using TMAO as a safety biomarker and this calls for further research.

## 4. TMAO as a Therapeutic Target

**Figure 2:**
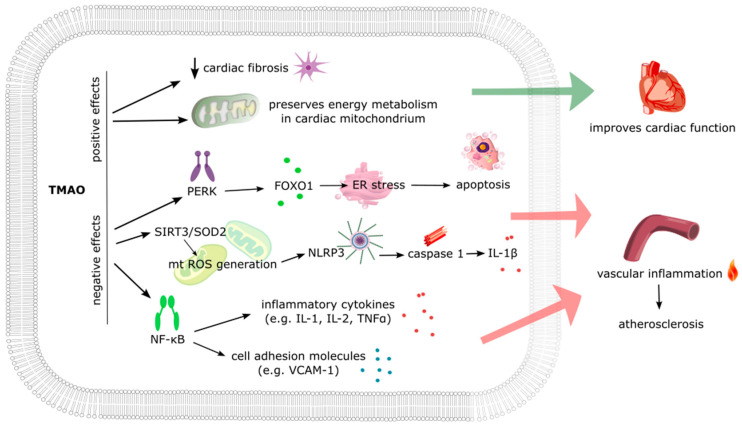
Hypothetical TMAO effects on cardiovascular pathology. ER stress—endoplasmic reticulum stress; FOXO1—Forkhead box protein O1; IL—interleukin; mt ROS—mitochondrial reactive oxygen species; NF-κB—nuclear factor kappa-light-chain-enhancer of activated B cells; NLRP3—NOD-, LRR-, and pyrin domain-containing protein 3; PERK—protein kinase R-like endoplasmic reticulum kinase; TMAO—trimethylamine N-oxide; TNFα—tumor necrosis factor alpha; VCAM-1—vascular cell adhesion molecule 1.

The role of TMAO in disease pathology, beyond its potential as a biomarker, remains a subject of debate (Figure 2). Animal studies have supported TMAO’s detrimental role by demonstrating various adverse effects following supplementation of TMAO or its dietary precursors [131]. Notably, TMAO intake has been linked to the exacerbation of heart failure [109,132,133], atherosclerosis development [134], impaired glucose tolerance [135], and liver dysfunction [136]. Dietary TMAO also increased platelet hyperreactivity and thrombosis risk [137], with evidence indicating that microbial CutC-dependent TMA production is sufficient to induce these effects [138]. There are several potential mechanisms of TMAO’s detrimental role. TMAO may promote vascular inflammation via NLRP3 inflammasome activation, which is partly mediated through the inhibition of the SIRT3–SOD2–mitochondrial ROS signaling pathway [139]. In this regard, TMAO has also been shown to impair β-oxidation and pyruvate metabolism in cardiac mitochondria [140]. It has been demonstrated that TMAO accelerate endothelial dysfunction, including decreasing endothelial self-repair and increasing monocyte adhesion via PKC/NF-κB/VCAM-1 [141,142]. In addition, TMAO’s pathological role in atrial fibrillation has been raised. Namely, TMAO, by activating p65 NF-κB signaling, altered the stability of atrial electrophysiology in normal canines and aggravated acute electrical remodeling in an atrial fibrillation canine model [143].

However, contrasting studies challenge TMAO’s pathological role [144]. Aldana-Hernández et al. found no association between TMAO and atherosclerosis development [145] and some research suggests a protective effect of TMAO precursors against atherosclerotic lesions [146]. Some studies indicate that TMAO supplementation leads to reduced fibrosis in the heart [147] and reduced mortality in heart failure rats alongside diuretic, natriuretic, and hypotensive effects [148]. Videja et al. suggest that TMAO accumulation in cardiac tissue improves mitochondrial energy metabolism [149]. In vitro studies present mixed results: while TMAO induces inflammation and endothelial dysfunction [139,150], it appears to be non-harmful to cardiomyocytes [151] or vascular smooth muscle cells [152]. These inconsistent findings leave TMAO’s role in cardiovascular pathology uncertain.

In contrast, TMA, the direct precursor of TMAO, has long been recognized for its toxic properties. As an industrial pollutant, TMA can cause various harmful effects, including eye or skin irritation, developmental toxicity, and neurological disorders at relatively low levels [153,154,155,156]. Surprisingly, its role in cardiovascular pathology is largely unexplored, though evidence suggests that TMA, not TMAO, may be the mediator in cardiovascular issues [129,157,158].

Targeting microbial-dependent TMA synthesis holds promise as a therapeutic approach. Reducing intestinal TMA production through antibiotics [7,133], probiotics [159], or non-lethal TMA lyase inhibitors [134,160,161,162,163] has shown potential. Studies confirm that inhibiting bacterial TMA production can mitigate atherosclerosis and related lipid disorders [134,159] and improve cardiac function [164,165]. These findings suggest TMAO as a modifiable risk factor, although further research, particularly in humans, is essential for conclusive evidence.

## 5. Future Directions

The burgeoning interest in TMAO research over the past decade underscores the necessity for high-quality data evaluating its potential as a biomarker. Despite the exponential growth in studies focusing on TMAO, its integration into general clinical practice for disease management and patient outcome improvement remains a distant goal. A significant gap in our current understanding involves the biological role of TMAO, which requires consensus and comprehensive investigation. Further research is essential to determine whether TMAO could serve as a superior marker compared to established cardiovascular markers like NT-proBNP or eGFR, particularly in specific patient populations. This necessitates comparative studies that not only measure the predictive accuracy of TMAO against these traditional markers but also explore its utility across diverse patient demographics, including those with varying cardiovascular risk profiles and comorbidities. Such investigations would help clarify the contexts in which TMAO could provide additional or enhanced prognostic value, potentially leading to more personalized and effective cardiovascular disease management strategies.

## Figures and Tables

**Table 1:** TMAO as a diagnostic biomarker in disease detection.

Detected Disease	Diagnostic Accuracy	Proposed TMAO Cutoff (μmol/L)	Reference	Comments
Acute ischemic stroke	Moderate (AUC = 0.75)	6.6	[15]	No adjustment to GFR
	Moderate (AUC = 0.78)	0.014	[38]	Unexpectedly low cut-off value
	Moderate (AUC = 0.729)	4.95	[17]	No adjustment to GFR
Atrial fibrillation	No differences between AF and healthy		[37]	
Coronary artery disease	Low (AUC = 0.6)	Not provided	[18]	
	Low (AUC = 0.56)	Not provided	[24]	No adjustment to GFR
	No differences between CAD and healthy		[36]	
Diabetic kidney disease	Low/moderate (AUC 0.691)	227	[39]	
Heart failure	Moderate (AUC = 0.881)	Not provided	[20]	
	Moderate (AUC = 0.817)	Not provided	[21]	HFpEF
	Low (AUC = 0.63)	0.094	[19]	HFpEF; no adjustment to GFR

Footnote. High/moderate/low accuracy by receiver operator characteristics (ROC) curves [Fisher 2003]; AF—atrial fibrillation; AUC—area under ROC curve; CAD—coronary artery disease; HFpEF—heart failure with preserved ejection fraction; GFR—glomerular filtration rate; HF—heart failure.

**Table 2:** TMAO as a prognostic biomarker.

Disease	Prognosed Outcome	Prognostic Accuracy	Median/Mean Follow-Up Duration	Reference	Comments
Peripheral artery disease	Mortality	HR 2.06	5 years	[43]	
	MACE, Mortality	OR 1.68	4 years	[22]	
Chronic kidney disease	MACE	HR 1.23	3 years	[58]	
	Mortality	HR 4.32	5 years	[59]	
	Mortality, MACE	No association	5.3 years (max)	[60]	
	Mortality	HR 1.93	5 years	[61]	
	CV Mortality, Mortality	HR 1.13; HR 1.14	6.1 years	[62]	Patients on hemodialysis
	MACE	AUC = 0.68	2 years	[63]	Patients on hemodialysis
	MI, Stroke, or Peripheral Artery Disease Event	No association	3.5 years	[64]	Patients with diabetes
	Mortality, CV Mortality	SHR 1.22; SHR 1.41 (Men only)	5.3 years	[65]	Patients on peritoneal dialysis
Hypertension	Stroke	OR 1.22	4.5 years	[66]	
Diabetes	Mortality	HR 2.7	4.8 years	[67]	
	MACE	No association	3.5 years	[68]	Type 2 diabetes with atherosclerosis risk factors
	MACE, Mortality	HR 2.05; HR 2.07	3 and 5 years	[69]	
	Mortality, CV Mortality, MACE	No association	6.8 years; 6.8 years; 6.5 years	[70]	
	MACE, Mortality	HR 1.29; HR 1.16	7.1 years	[71]	Type 2 diabetes
Carotid atherosclerosis	CV Mortality	No association	5.3 years	[51]	
Acute coronary syndrome	Mortality	HR 1.81	7 years	[72]	
	MACE	No association	6.7 years	[54]	No association after adjustment to GFR
	Mortality	No association	5 years	[55]	
	MACE	HR 1.73	1 year	[73]	Patients on anti-platelet therapy
	MACE	HR: 1.85	2 years	[74]	Acute myocardial infarction and heart failure
	CV Mortality, Stroke	OR 1.89; OR 2.01	33 months	[75]	Prior myocardial infarction
	MACE	HR 1.59	2 years	[76]	High TMAO levels at both time points; no adjustment to GFR
	CV Mortality	HR 11.62	7 years	[42]	
	MACE	HR 2.61	1 year	[77]	STEMI
	New-Onset AF	OR 1.29	1 year	[78]	No adjustment to GFR
	Left Ventricular Systolic Dysfunction	No association	30 days	[79]	STEMI
Acute ischemic stroke	MACE	HR 3.3	1 year	[80]	
	Poor Functional Outcome, Mortality	OR 3.09; OR 5.64	3 months	[81]	
	Poor Functional Outcome, Mortality	OR 1.21; OR 1.36	3 months	[31]	
	MACE	HR 1.69	1.9 years	[82]	
	Post-Stroke Cognitive Impairment	OR 3.30	1 year	[83]	
	Stroke Recurrence, MACE	HR 1.37	1 year	[84]	
	Stroke Recurrence	HR 1.28	1 year	[85]	
	Functional Outcome	No association	At discharge	[86]	
	MACE	HR 3.128	3 month	[38]	
	Major Ischemic Event, Poor Functional Outcome	OR 3.59; OR 2.58	1 year	[47]	
Coronary artery disease	Mortality	HR 1.95	5 years	[44]	
	Acute Myocardial Infarction	No association	4.9 years	[87]	Stable angina pectoris
	Mortality, CV Mortality	HR 1.58; HR 1.66	5 years	[88]	
	Mortality, CV Mortality	No association	9.8 and 10.5 years	[89]	Two cohorts: CAD and community-based adults
	MACE	log rank *p* = 0.004	1.5 years	[90]	Stable angina pectoris
Individuals with and without CV disease	Cardiovascular Mortality, Mortality	HR 1.8; HR 1.9	9.7 years	[91]	
Heart failure	Mortality or HF Hospitalization	HR 3.82	5 years	[46]	HF with preserved EF
	Mortality/Heart Tx	HR 1.46	5 years	[92]	
	MACE, Mortality	HR 1.57; HR 1.53	1.8 years	[93]	
	Cardiovascular Mortality, HF Hospitalization	HR 2.03; HR 1.96	2.4 years	[94]	HF with preserved EF
	Mortality, Mortality/HF	HR 1.26; 1.25	1 year	[40]	
Sever aortic stenosis	Mortality	HR 1.79	4.2 years	[95]	
Atrial fibrillation	Mortality, CV Mortality, Stroke;	HR 1.65 HR 1.86 No association	4 years	[96]	

Footnote. AF—atrial fibrillation; CAD—coronary artery disease; EF—ejection fraction; GFR—glomerular filtration rate; HF—heart failure; HR—hazard ratio; MACE—major adverse cardiovascular events; OR—odds ratio; Tx—transplantation.
